# Patients with pemphigus are at an increased risk of developing rheumatoid arthritis: a large-scale cohort study

**DOI:** 10.1007/s12026-020-09160-6

**Published:** 2020-11-07

**Authors:** Khalaf Kridin, Virginia A. Jones, Payal M. Patel, Shira Zelber-Sagi, Christoph M. Hammers, Giovanni Damiani, Kyle T. Amber, Arnon D. Cohen

**Affiliations:** 1 Lübeck Institute of Experimental Dermatology, University of Lübeck, Ratzeburger Allee 160, 23562 Lübeck, Germany; 2 Department of Dermatology, University of Illinois at Chicago, Chicago, IL USA; 3 University of Haifa, Haifa, Israel; 4 Clinical Dermatology, IRCCS Istituto Ortopedico Galeazzi, 20161 Milan, Italy; 5 Clalit Health Services, Tel-Aviv, Israel

**Keywords:** Rheumatoid arthritis, Pemphigus, Cohort study

## Abstract

Data regarding the association between pemphigus and rheumatoid arthritis (RA) is inconclusive and yet to be firmly established. In the current study, we aimed to evaluate the risk of developing RA during the course of pemphigus. A large-scale population-based longitudinal cohort study was conducted to evaluate the hazard ratio (HR) of RA among 1985 patients with pemphigus relative to 9874 age-, sex-, and ethnicity-matched control subjects. A multivariate Cox regression model was utilized. The incidence of RA was 1.07 (95% CI, 0.62–1.72) and 0.36 (95% CI, 0.24–0.52) per 1000 person-years among patients with pemphigus and controls, respectively. The lifetime prevalence of RA was 2.3% (95% CI, 1.7–3.1%) among cases and 1.8% (95% CI, 1.5–2.0%) among controls. Patients with pemphigus were more than twice as likely to develop RA as compared to control subjects (adjusted HR, 2.54; 95% confidence interval [CI], 1.31–4.92). The increased risk was robust to a sensitivity analysis that included only cases managed by pemphigus-related systemic medications (adjusted HR, 2.56; 95% CI, 1.30–5.05). In conclusion, pemphigus is associated with an increased risk of RA. Physicians treating patients with pemphigus should be aware of this possible association. Further research is required to better understand the mechanism underlying this association.

## Introduction

Pemphigus encompasses a group of rare, potentially lethal, mucocutaneous autoimmune bullous dermatoses [1]. These intraepidermal bullous diseases manifest with vesicles and erosions on the epithelium of mucous membranes and skin, often causing a substantial impairment of quality of life and elevated morbidity and mortality [2, 3]. The pathogenesis results from the production of IgG autoantibodies against epidermal cadherins [4, 5]. It is well studied that autoimmune disease, such as pemphigus, is likely to coexist within individuals and their relatives [6–8]. Several studies have substantiated the concept of autoimmune diathesis in pemphigus with an array of autoimmune diseases [1].

Rrheumatoid arthritis (RA) is a chronic, usually symmetric, inflammatory autoimmune disease, primarily afflicting peripheral joints [9]. While the exact etiology of RA is not fully understood, environmental factors such as smoking, in a genetically prone individual, are believed to the major stimuli in RA development [9]. A high burden of autoimmune disease had been observed among patients with RA, with 24% of patients having at least one concomitant autoimmune disease [10].

A descriptive cluster analysis has demonstrated that pemphigus forms a distinct cluster with (RA autoimmune thyroid diseases (AITD), and type I diabetes mellitus[11]. In the meanwhile, two other observational studies did not reveal a significant association between pemphigus and RA [3, 12]. Of interest, a reciprocal human leukocyte antigen (HLA) disease association between the HLA-DRB1 alleles for pemphigus vulgaris (PV) and RA has been established in the literature [13]. While HLA-DRB1*04:02 allele confers a predisposing risk to PV, it is protective in RA [13, 14]. Taken together, the association between these conditions is inconclusive and yet to be firmly established both epidemiologically and genetically.

The aim of the current study is to further explore the association between pemphigus and RA by conducting a large-scale retrospective cohort study investigating the risk of developing RA among patients with pemphigus.

## Methods

### Study design and database

We conducted a retrospective cohort study to gauge the risk of developing incident RA among patients with pemphigus compared to control individuals.

The information for this study was obtained from the computerized dataset of Clalit Healthcare Services (CHS). CHS is the largest Israeli health organization, providing both public and semi-private healthcare services to approximately 4,400,000 insured patients as of 2016 (which is approximately 54% of Israel’s general population consistent with the 2016 census). CHS allows research professionals to automatically retrieve and extract patient data for epidemiological studies by use of a comprehensive computerized database with constant real-time input from medical, pharmaceutical, and administrative operating systems.

Additionally, CHS offers a chronic disease registry which collects data from different sources, including those of health systems, primary care physicians, and specialists, and are regarded as vastly reliable[15].

### Study population and covariates

Pemphigus and RA disease diagnoses were defined by the following criteria: a documented diagnosis of these entities, twice, as a minimum, in the medical records as documented by a community physician, or when hospital discharge documentation recorded the diagnoses. The control group consisted of up to 5 controls per patient, matched at random by age, gender, and ethnicity. Age matching was based on the exact year of birth (1-year strata). Diagnosis date was used as an index date for the cases and each matched control.

A sensitivity analysis aiming to increase the validity of the diagnosis was performed. The latter repeated all calculations after including only cases prescribed “pemphigus-related medications”: systemic corticosteroids or adjuvant immunosuppressive agents (azathioprine, mycophenolate mofetil, or cyclophosphamide) for more than 6 months; or cases prescribed one or more cycles of rituximab.

Outcome measures were controlled for healthcare overutilization in order to rule out ascertainment bias in our observed associations. Healthcare utilization was defined by each individual’s total visit number in the year prior to the diagnosis of pemphigus or the date of enrollment for control subjects. Additional adjustment for comorbid conditions was performed by use of the Charlson comorbidity index; a validated method of measuring comorbidity which has been shown to be a dependable predictor of lethal outcomes [16].

### Statistical analysis

Baseline characteristics are described by means and standard deviations (SDs) for continuous variables, whereas frequencies and percentages are used to describe categorical values. A comparison of the distribution of sociodemographic and clinical factors between patients with and without pemphigus was conducted, where the chi-square test was used to describe sex and socioeconomic status, and *t* test was used for age.

Incidence rates of RA were calculated for both pemphigus patients and controls and expressed as the number of events per 1000 person-years. Hazard ratios (HRs) for the risk of incident RA were obtained by use of Cox regression models, whereas odds ratio (OR) were calculated through logistic regression. The incidence of RA during follow-up was calculated only for individuals without a history of RA before study initiation. The cumulative incidence of RA was compared between pemphigus and control groups using a stratified log-rank test. Two-tailed *P* values less than 0.05 were considered statistically significant, whereas results with 95% confidence intervals (CIs) were reported where applicable. SPSS software, version 25 (SPSS, Chicago, IL, USA) was utilized to perform all statistical analyses.

## Results

A total of 1985 patients with pemphigus, diagnosed between 2004 and 2014 and 9874 age-, sex-, and ethnicity-matched control subjects were included in the study. No significant difference was found between the average age with, sex distribution, ethnicity, and socioeconomic status of the two groups; the mean (± SD) age of study participants was 72.1 ± 18.5 years and 40.2% were males. The Charlson comorbidity index rate was higher in pemphigus patients, with 1059 (53.4%) patients affected by severe comorbidity compared to 4055 (41.1%) control subjects (*P* < 0.001; Table 1).

**Table 1:** Descriptive characteristics of the study population

Characteristic	Patients with pemphigus (*N* = 1985)	Controls (*N* = 9874)	*P* value
Age, years			
Mean ± SD	72.1 ± 18.5	72.1 ± 18.5	1.000
Median (range)	77.4 (0–103.0)	77.4 (0–103.1)	
Male sex, *N* (%)	797 (40.2%)	3962 (40.1%)	0.934
Ethnicity, *N* (%)			
Jews	1805 (90.9%)	8866 (89.8%)	0.136
Arabs	180 (9.1%)	1008 (10.2%)	
BMI, kg/m^2^ (mean ± SD)	27.7 ± 6.6	27.9 ± 6.6	0.355
Smoking, *N* (%)	510 (25.7%)	2758 (27.9%)	0.045
SES, *N* (%)			
Low	634 (31.9%)	3249 (32.9%)	0.386
Intermediate	830 (41.8%)	4263 (43.2%)	0.250
High	423 (21.3%)	2217 (22.5%)	0.241
Charlson comorbidity score, *n* (%)			
None (0)	344 (17.3%)	2636 (26.7%)	< 0.001
Moderate (1–2)	582 (29.3%)	3183 (32.2%)	0.011
Severe (≥ 3)	1059 (53.4%)	4055 (41.1%)	< 0.001

*Abbreviation*s: *N*, number; *SD*, standard deviation; *BMI*, body mass index; *SES*, socioeconomic status

The lifetime prevalence of RA was slightly higher among patients with pemphigus (2.3%; 95% CI, 1.7–3.1%) as compared to controls (1.8%; 95% CI, 1.5–2.0%), although it did not exceed the level of statistical significance (OR, 1.33; 95% CI, 0.96–1.85). In cases where RA preceded the onset of pemphigus, the mean latency from RA to pemphigus was 8.2 ± 4.0 years, whereas when pemphigus preceded the onset of RA, the mean latency to RA was 3.4 ± 3.3 years.

During the follow-up period, a total of 15 and 25 patients developed new-onset RA, among the case and control groups, respectively. The total follow-up time was 14,046.0 person-years for patients with pemphigus and 70,378.5 person-years for controls. Taken together, the incidence of RA was 1.07 (95% CI, 0.62–1.72) and 0.36 (95% CI, 0.24–0.52) per 1000 person-years among patients with pemphigus and controls, respectively (Table 2).

**Table 2:** Incidence rates and hazard ratio of new-onset rheumatoid arthritis among patients with pemphigus (cohort study design)

	Patients with pemphigus	Controls
Follow-up time, PY	14,046.0	70,378.5
Median follow-up time, years (range)	6.9 (0.0–13.0)	6.9 (0.0–13.0)
Number of events	15	25
Incidence rate/1000 PY	1.07	0.36
95% CI	0.62-1.72	0.24-0.52
	HR (95% CI)	*P* **value**
Crude	*3.00* (*1.58–5.69*)	*0.001*
Adjusted*	*2.54* (*1.3–4.92*)	*0.006*
Sensitivity analysis**		
Crude	*3.30* (*1.70–6.45*)	< *0.001*
Adjusted*	*2.56* (*1.30–5.05*)	*0.007*

*Abbreviations*: *HR*, hazard ratio; *CI*, confidence interval; *PY*, person-year

*Following the adjustment for age, sex, ethnicity, socioeconomic status, comorbidities, and healthcare utilization

**Sensitivity analysis included only pemphigus patients under prolonged “pemphigus-specific treatments”

Italic: significant value

**Fig. 1:**
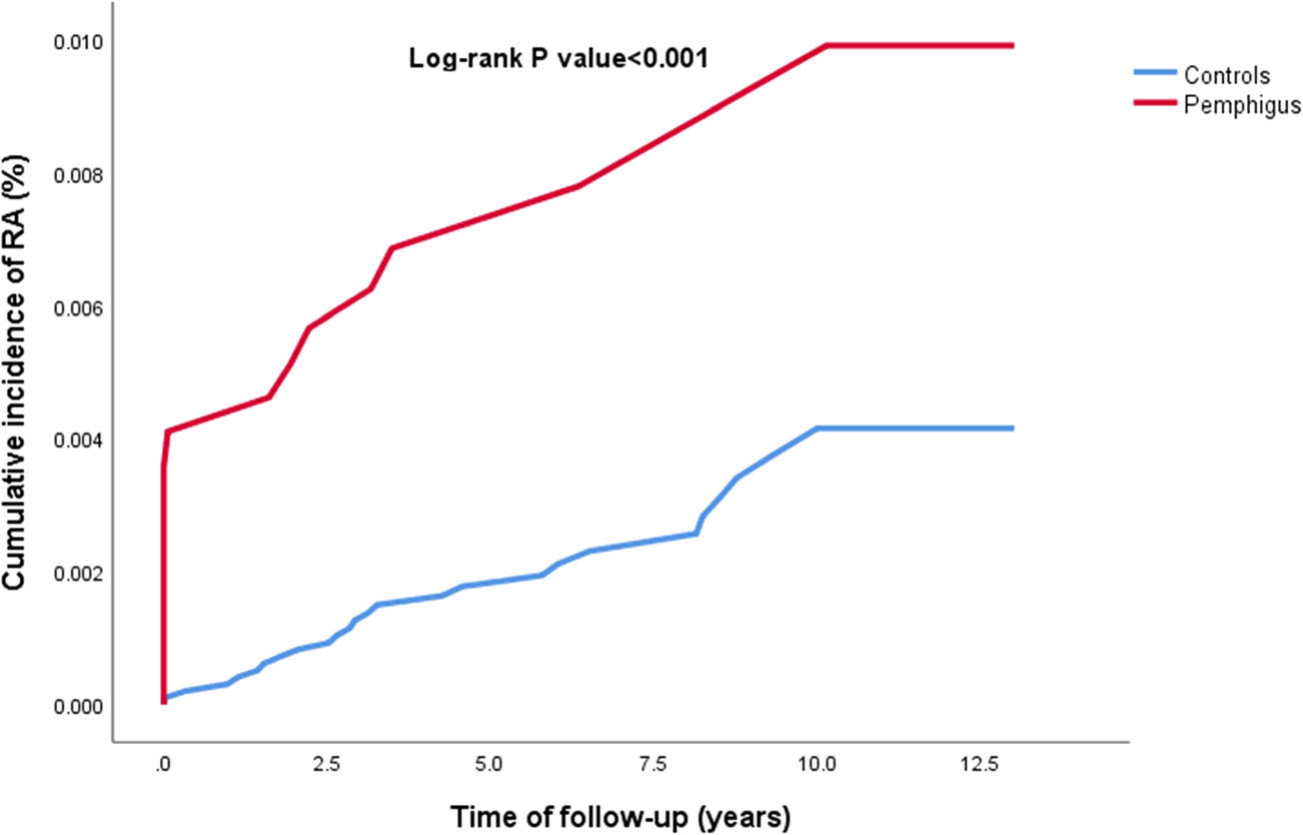
The cumulative incidence of RA among patients with pemphigus and controls

The crude risk of RA was threefold higher in patients with pemphigus than in control individuals (HR, 3.00; 95% CI, 1.58–5.69; Fig. 1). In a subgroup analysis, the crude risk was increased in female (HR, 3.34; 95% CI, 1.70–6.58), but not in male (HR, 1.25; 95% CI, 0.14–11.17) patients. After adjusting for several confounding factors, including demographic features, healthcare utilization, and comorbidities, pemphigus remained an independent significant risk factor for incident RA (adjusted HR, 2.54; 95% CI, 1.31–4.92; Table 2).

We then conducted a sensitivity analysis including solely patients with pemphigus who were prescribed “pemphigus-related treatments.” The crude risk of RA had been substantiated (HR, 3.30; 95% CI, 1.70–6.45), whereas the adjusted risk was not altered substantially following the adjustment for several putative confounding factors (adjusted HR, 2.56; 95% CI, 1.30–5.05; Table 2)

Of note, a diagnosis of both pemphigus and RA does not significantly increase the risk of all-cause mortality relative to the mortality observed among patients with isolated pemphigus (HR, 1.31; 95% CI, 0.91–1.87).

## Discussion

The current retrospective cohort study demonstrates that pemphigus patients are at more than twofold increased risk of developing RA as compared to control subjects (HR, 2.54). The results revealed that the risk of RA was particularly elevated in females (HR, 3.34), a finding that accords with the female preponderance observed in several autoimmune diseases [17, 18].

### The association between pemphigus and RA in the previous literature

Based on the findings of well-designed epidemiological studies, pemphigus has been reported to associate with other autoinflammatory and autoimmune comorbid disorders, such as RA [11, 19], ulcerative colitis [20], AITD [11, 19, 21], and psoriasis [3, 12, 22, 23]. Likewise, patients with RA experience a high burden of comorbid autoimmune conditions. A large-scale cross-sectional study had found that 24.3% and 6.0% of patients with RA have at least one and more than one comorbid autoimmune disease, respectively, with ankylosing spondylitis and psoriatic arthritis being most frequently encountered [10]. These observations are perceived as part of the concept known as “autoimmune diathesis,” in which individuals affected by an autoimmune disease are more vulnerable to acquire other autoimmune disease [6–8].

Leshem et al. [19] and Parameswaran et al. [11] revealed that the prevalence of RA was significantly greater among patients with pemphigus than in their first-degree relatives and the general population, respectively. Both studies, however, were uncontrolled and small scale, and the latter study relied on surveys and patient-reported outcomes. In contrast, a case-control study from Taiwan [12] and a cross-sectional study from the USA [3] failed to demonstrate a significant association between the two conditions. While the association between pemphigus and RA had been evaluated in the aforementioned studies [3, 11, 12, 19], the incidence of RA among patients with pemphigus, as well as the risk of RA during the course of pemphigus, is yet to be investigated.

### Putative interpretation for the study findings

The mechanism responsible for the association between RA and pemphigus is yet to be determined, but immunogenetic involvement has been speculated. Immunogenetic studies show an important association between susceptibility to PV and HLA-DRB1*04:02, primarily in Ashkenazi Jews, and HLA-DRB1*14:01, HLA-DRB1*14:04, and HLA-DQB1*05:03, prevalent in non-Jewish patients of European and Asian descent [24]. Yet, HLA-DRB1*04:02 is protective in RA, as it lacks the “shared epitope” seen in several high-risk HLA allotypes in RA. Notably, this shared epitope is also missing from DRB1*13:01, DRB1*13:02 [13]; thus, HLA DRB1*04:02 does not appear to be a shared risk factor in our population. We hypothesize that alternative T cell regulatory pathways may explain this shared autoimmune phenomenon in the presence of contradictory data for DRB1*04:02 in both diseases [25].

Interleukin 17 (IL-17) is a pro-inflammatory cytokine produced by Th17 cells which serves as a bridge between innate and adaptive immunity [26, 27]. Evidence supports an association between RA and IL-17 elucidating that excessive IL-17 receptor signaling is an essential pathway in converting an acute synovitis into the chronically damaging arthritis seen in RA [26–28]. Likewise, a recent meta-analysis has identified vastly increased levels of serum IL-17 in patients with pemphigus, perhaps expounding on the association between pemphigus and RA [29].

### Strengths and limitations

While the current study is the first cohort study to shed light on the association between pemphigus and RA, and to contributes a novel epidemiological feature regarding the risk of developing RA during the course of pemphigus, it is not without limitations. The management of pemphigus patients generally includes high doses of corticosteroids and to immunosuppressive agents [30–32], which should theoretically improve subclinical RA or mask any underlying symptoms [33]. We speculate that this may lead to an underestimation in the incidence of RA among patients with pemphigus due to underreported diagnosis of RA. The diagnosis of cases with pemphigus and RA was not made on the basis of histologic or immunopathological criteria, and the clinical characteristics of eligible cases could not be retrieved. However, previous studies had demonstrated that the chronic registry of CHS was proven very reliable.

In conclusion, we demonstrate an increased risk of developing RA among female pemphigus patients. Prospective studies are needed to evaluate this epidemiological relationship in other ethnic groups. In pemphigus patients presenting with concerning symptoms of RA, additional investigation may be warranted.
